# Cancer recurrence and lethality are enabled by enhanced survival and reversible cell cycle arrest of polyaneuploid cells

**DOI:** 10.1073/pnas.2020838118

**Published:** 2021-01-27

**Authors:** K. J. Pienta, E. U. Hammarlund, J. S. Brown, S. R. Amend, R. M. Axelrod

**Affiliations:** ^a^The Brady Urological Institute, Johns Hopkins School of Medicine, Baltimore, MD 21287;; ^b^Translational Cancer Research, Department of Laboratory Medicine, Lund University, 223 81 Lund, Sweden;; ^c^Nordic Center for Earth Evolution, University of Southern Denmark, 5230 Odense, Denmark;; ^d^Cancer Biology and Evolution Program, Moffitt Cancer Center, Tampa, FL 33612;; ^e^Department of Integrated Mathematical Oncology, Moffitt Cancer Center, Tampa, FL 33612;; ^f^Gerald R. Ford School of Public Policy, University of Michigan, Ann Arbor, MI 48109

**Keywords:** metastasis, drug resistance, tumor microenvironment, whole-genome doubling, evolution

## Abstract

We present a unifying theory to explain cancer recurrence, therapeutic resistance, and lethality. The basis of this theory is the formation of simultaneously polyploid and aneuploid cancer cells, polyaneuploid cancer cells (PACCs), that avoid the toxic effects of systemic therapy by entering a state of cell cycle arrest. The theory is independent of which of the classically associated oncogenic mutations have already occurred. PACCs have been generally disregarded as senescent or dying cells. Our theory states that therapeutic resistance is driven by PACC formation that is enabled by accessing a polyploid program that allows an aneuploid cancer cell to double its genomic content, followed by entry into a nondividing cell state to protect DNA integrity and ensure cell survival. Upon removal of stress, e.g., chemotherapy, PACCs undergo depolyploidization and generate resistant progeny that make up the bulk of cancer cells within a tumor.

It is widely recognized that the majority of cancer cells within a tumor have an abnormal number of chromosomes, i.e., are aneuploid (1–4). A polyaneuploid cancer cell (PACC) is an aneuploid cancer cell that has undergone whole-genome doubling (WGD), resulting in at least twice the complement of the original aneuploid genomic content. Unusually large aneuploid cancer cells have been documented in the cancer literature since 1858 when they were first described by Virchow (5–38). These giant aneuploid cells, observed in cell culture and pathologic tissues from patients, have been generally disregarded as not functionally important: irreversibly senescent or destined for mitotic catastrophe, too morphologically misshapen, and with chromatin too disorganized to directly contribute to tumorigenesis.

Recent evidence demonstrates that PACCs are viable and exist as a distinct and functional cancer cell state, able to resist stress within the tumor microenvironment (5–31). This allows us to develop a unifying theory to explain cancer recurrence and lethality that builds upon and unifies the observations of multiple fields of study, including aneuploidy, stem cell biology, genetic instability, tumor cell heterogeneity, senescence, quiescence, therapeutic resistance, and giant cells. The “hallmarks of cancer” provide the framework for tumorigenesis, but the hallmarks do not explain therapeutic resistance, recurrence, or lethality (39). The basis of our theory is that the formation of the PACC state in response to microenvironmental and therapeutic stress enables resistance to systemic cancer therapy

Metastatic cancer is ultimately resistant to virtually all systemic therapies and continues to kill more than 10 million people per year around the world (40–42). This suggests a common mechanism for cancer resistance that evolves convergently in 10 million people each year, regardless of the driver mutation or the tissue of origin (42, 43). Resistance to therapeutic interventions has classically been attributed to genetic tumor cell heterogeneity: Among the billions of cancer cells in a tumor, mutations lead to at least one cancer cell becoming resistant to a particular therapy (44–65). Since lethal cancer demonstrates resistance to therapeutic agents that it has previously not been exposed to, particular resistance mutations appear to develop by stochastic chance, fueled by aneuploidy (an abnormal number of chromosomes) and genetic instability (32, 44, 60, 61, 66–68). In the classic view, resistance to each different therapy requires that the appropriate mutations that confer the different versions of resistance are acquired by at least one cell. Newer models have found potential evidence for the gradual, multifactorial adaptation to the inhibitors through acquisition of multiple cooperating genetic and epigenetic adaptive changes of multiple partially resistant clones (69). Another alternative model of therapy resistance is the cancer stem cell model, in which a rare therapy-resistant population of cancer stem cells give rise to a recurrent population (36, 70–72).

In contrast, we theorize that lethal cancer is mediated through the generation of PACCs. PACCs form by utilizing evolutionary and developmental programs that utilize variations of the canonical cell cycle that allow bypass of mitosis and/or cytokinesis and permit entry into a polyploid state (73–78). These polyploidization programs are commonly observed as transient defense mechanisms in other organisms and in human tissue as an adaptation to environmental or metabolic stressors. For example, evolutionary programs for polyploidization have for eons enabled both unicellular and multicellular organisms a transient defense against toxins by entry into a nonproliferative state. Over the evolutionary history of life, polyploid programs have led to several events of persistent WGD, i.e., species polyploidy, in which genetic alterations associate with survival adaptations. The PACC theory of therapeutic resistance and cancer recurrence accounts for the ubiquity of recurrence in three steps (Fig. 1). First, a few tumor cells respond to stress (e.g., chemotherapy or metabolic stress as a consequence of uncontrolled growth) by accessing alternate cell cycle programs that form PACCs. Second, as part of cell enlargement and the polyploid program, a PACC pauses cell division, which allows it to adapt to toxic environments while protecting its DNA. This state also provides increased cell resiliency and survival in foreign secondary sites, enabling successful metastatic seeding. Third, when the stressor is removed, the PACC can undergo depolyploidization and reinitiate cancer cell proliferation. The resulting cells resume the cell cycle and now carry novel adaptations for overcoming the host defenses as represented in the hallmarks of cancer (Fig. 1).

**Fig. 1.**
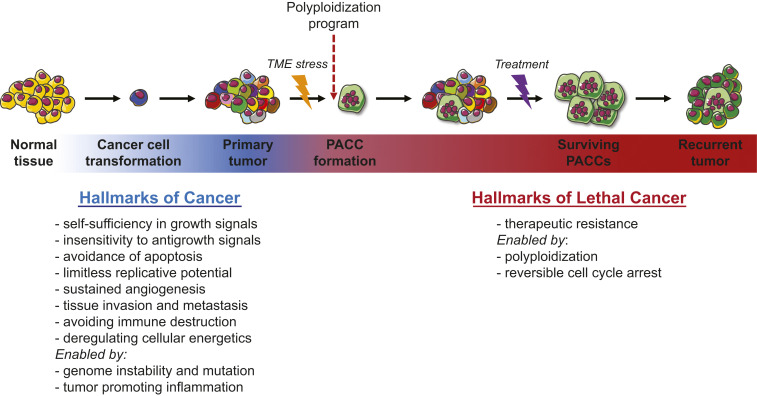
The ability to access polyploid programs enables therapeutic resistance, the hallmark of lethal cancer. The PACC theory of cancer recurrence accounts for the ubiquity of recurrence in three steps. First, a few tumor cells respond to stress, e.g., chemotherapy or metabolic stress as a consequence of uncontrolled growth, through a polyploid program and form PACCs. Second, as part of cell enlargement and the polyploid program, PACCs pause proliferation, allowing adaptation to toxic environments while protecting DNA. This state is also associated with motility, which further enables metastasis. Third, when the stimulus is a therapy and is removed, the PACC can undergo depolyploidization, reinitiating tumor cell proliferation and recurrence.

Cells that have the capacity to form PACCs can alter their cell cycle to bypass mitosis and/or cytokinesis to become polyploid and are therefore not shunted to apoptosis and consequently exhibit enhanced survival under conditions of stress (79). The associated increase in cell size and concomitant decrease in surface-to-volume ratio may either protect the cell from local environmental damage by limiting the overall toxin load within the cell or assist the cell to broaden its environment for the scavenging of oxidants and nutrients. Simultaneously, the increase in genomic material resulting from polyploidization may provide building blocks for increased RNA and protein synthesis that provide raw materials for increased cellular metabolism, detoxification, and extended dormancy. In addition, the increase in genomic material may enable the cell to either avoid lethal genomic damage secondary to extra copies of genes, increase heritable variation, or allow self-genetic modification that allows new functionality or selection of robust progeny.

## PACCs

**Fig. 2.**
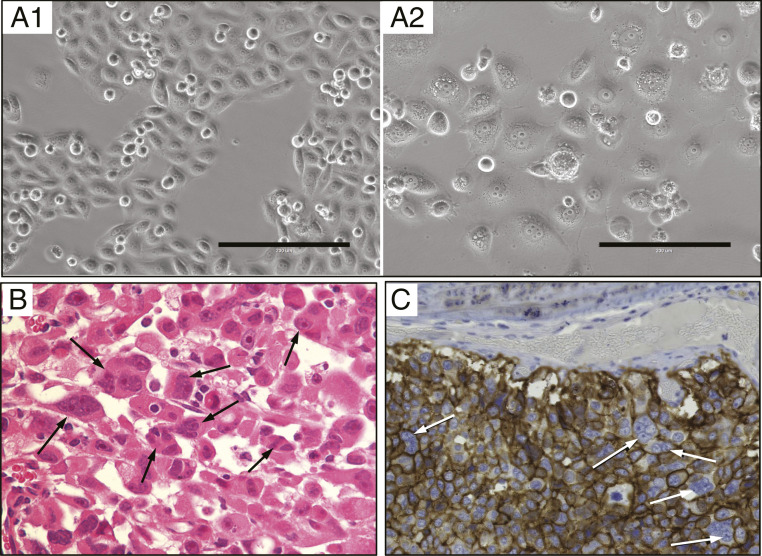
Polyaneuploid cancer cells (PACCs). PACCs are observed in many cell lines. Prostate cancer cell line PC3 as an example before (*A.1*) and at 72 h after treatment with 10 nM docetaxel (*A.2*). They are also found in animal models (*B*: PC3 xenograft, 200,000 PC3 cells were injected s.c. in an NSG mouse; tumors were harvested on day 21 and processed for H&E [PACCs circled]) and in patients (*C*: lung metastasis from a patient with castrate-resistant prostate cancer stained with EpCAM).

PACCs exist as a distinct state of viable cancer cells, unique in that they contain multiple full copies of their aneuploid genome (5–38). The terminology for these cells has varied, including polyploid giant cancer cells, multinucleated giant cancer cells, blastomere-like cancer cells, osteoclast-like cancer cells, pleomorphic cancer cells, large cancer stem cells, and PACCs. Growing evidence now suggests that PACCs are functional actuators of therapeutic resistance. Notably, PACCs are observed in cancer cell lines (Fig. 2 *A.1* and *A.2*), in animal models (Fig. 2*B*), and in patients (Fig. 2*C*) across virtually all tumor types (5–31). Our growing insight into the role of PACCs in tumor progression underscores the importance of defining their biology, function, and relevance to cancer lethality. Important open questions concern how and when PACCs arise during tumorigenesis, how PACCs serve an adaptive role for cancer cells, and how they contribute to cancer’s lethality.

Here, we offer a unifying theory for these three questions. Our theory relies on emerging data on how therapeutic resistance arises from PACCs. In this model, resistance is mediated through access to evolutionary and developmental programs for polyploidy that increase cell size and contents, including DNA. The increased cell size and organelle contents allow increased capacity for metabolism as well as for mitigating toxic stressors such as reactive oxygen species (63, 80, 81). Cancer cells with the ability to form PACCs exit the cell cycle and avoid DNA damage, potentially providing a universal mechanism of therapeutic resistance that is mutation (and therefore therapy) agnostic. This process shares many similarities with the phenomenon of therapy-induced reversible senescence (23, 26, 34, 36, 82–87). Further investigations are required to understand how cell cycle exit, quiescence, and senescence are related (87–89). While exiting the cell cycle and maintaining quiescence until the systemic stress has passed may be enough to allow repopulation of the 2N+ cancer cell population, access to greater genomic material could also mediate resistance in PACC progeny. The access to greater amounts of genetic material promotes genomic stability through extra copies of genes while simultaneously allowing for the potential generation of resistant progeny through either diverse mutations (e.g., gene duplication, chromosomal rearrangements) or via self-genetic modification prior to producing progeny. This access to increased heritable variation may be dispensed to their 2N+ aneuploid progeny that make up the bulk of cancer cells within a tumor, providing ecological and evolutionary rescue to otherwise-doomed populations (5, 16, 29, 35, 37, 39, 90–95). PACCs, therefore, share many similarities with cancer stem cells (16, 48, 96, 97). Defining the stem cell properties of PACCs and the mechanisms by which PACCs generate 2N+ progeny requires further investigation.

## Cancer Cells Access Evolutionary and Developmental Polyploid Programs to Form PACCs

Polyploid programs provide increased fitness both on an evolutionary timescale (across generations of organisms) and within the life span of a single organism. While the evolutionary programs can be acute responses to environmental anomalies or protection against mutation, such developmental programs are the norm for some specialized tissues. The evolutionary role precedes the developmental role of these programs as they arose in prokaryotes and single-celled eukaryotes long before the evolution of multicellular organisms. Cancer cells, as single-celled organisms and as decendants of a multicellular host, may engage either (or both) the evolutionary or developmental advantages of polyploidization. Understanding and applying these evolutionary and developmental programs may reveal the key stimuli that acutely and transiently induce polyploidy as well as reveal the benefits and costs of maintaining polyploid cells in cancer.

PACCs form in response to many different natural and synthetic environmental stimuli such as hypoxia, lack of nutrients, changes in pH, or chemotherapy (5–31, 38). Within a multicellular organism, somatic cells that demonstrate chromosomal anomalies are commonly shunted to apoptosis. Moreover, somatic cells that experience severe environmental deviations, such as abnormally low pH, are lysed. In contrast, PACCs exhibit a protected cellular phenotype that is largely indifferent to a changing environment, whether local (changing tumor microenvironment) or systemic (toxic therapy). This type of resilience to environmental disruption through polyploidy and cell enlargement has been noted throughout evolution from unicellular to multicellular organisms. If PACCs can access such an evolutionarily defined response program, their ability to survive therapeutic stress may simply be a by-product of this ancestral capacity (98). These evolutionary response programs of increasing cell size and DNA material are reflected in the developmental programs that are accessed by normal cells in response to stress. Once cancer cells mobilize this ability, they also gain the ability to survive and react to environmental stresses within the tumor microenvironment such as changes in oxygen, nutrients, and pH, i.e., the cancer swamp (99–101).

The formation of polyploid cells is observed across unicellular and multicellular eukaryotic organisms and is associated with distinct survival advantages (Table 1). Bacteria, protists (unicellular eukaryotic organisms), plants, fungi, invertebrate animals, and vertebrates demonstrate cell enlargement by polyploidy during development as well as temporal crises. Cells become polyploid through either cell fusion or more commonly by DNA duplication (102–106). DNA duplication occurs by alterations to the normal cell cycle through either mitotic slippage, endocycling (also termed endoreduplication or endoreplication), or endomitosis (cytokinesis failure) (105, 107–109). Mitotic slippage is a semicomplete cell cycle in which the cell fails to resolve the mitotic spindle assembly checkpoint and, after a delay, exits a prolonged mitosis with a 4N nucleus (110). Endocycling encompasses alternating gap and S-phases that can result in polyploid cells of varying ploidy, up to 1,000 copies of the genome (111). Many endocycling cells do not display early mitotic markers, such as nuclear envelope breakdown, and some exhibit truncated S-phases where late-replicating DNA in heterochromatic regions is not fully duplicated, leading to genomic deletions (76). Endomitosis is achieved by bypassing cytokinesis or late mitosis, resulting in multinucleate cells or lobulated nuclei when anaphase is not completed (e.g., the formation of megakaryocytes). The mitotic cycle and the endocycle that results in replication of the genome without complete mitosis are linked through tight regulation of the cell cycle program at multiple checkpoints (81, 104, 112–120). The failure of nuclear division, or endomitosis, can also be achieved in two ways. If only cytokinesis is blocked, multinucleated polyploid cells are formed, but if karyokinesis is inhibited, polyploid cells possess a single nucleus.

**Table 1.** Postulated consequences for polyploidization including whole-genome doubling

Consequence	Description
Genomics	
1. Increased genomic stability	Extra copies of genes allow organisms to avoid lethal genomic damage, e.g., preventing Muller’s ratchet in protists.
2. Increased heritable variation	The increased genomic material allows increased mutation in response to stress. Genetic instability creates progeny of various fitness allowing selection of a robust clone, e.g., antibiotic resistance in some yeast strains.
3. Self-genetic modification	Increased genomic material provides self-genetic modification through directed reprogramming, e.g., antibiotic resistance in some bacteria strains.
4. New functionality	Redundant genomic material allows mutation to achieve a new functionality. For example, two pairs of limbs allow one pair to become wings.
Function	
5. Induction of quiescence	Halting of the cell cycle leads to a nonproliferative state as a mechanism to protect the nondividing genome while stress is present, e.g., *Entamoeba histolytica*.
6. Increased storage capacity	Increased cell size increases storage capacity needed for sustained quiescence (genomic material is a passenger), e.g., plant vacuoles.
7. Increased cell function	Increased cell size increases cell function (genomic material is a passenger), e.g., osteoclast fusion for the production of acid to lyse bone.
8. Increased metabolic capacity	Increased gene dosage increases production of RNA and protein products necessary for increased cell metabolism for growth, e.g., megakaryocytes.
9. Increased toxin protection	Increased gene dosage increases production of RNA and protein products necessary to protect from oxidative damage and cell size may protect from short-term environmental toxic stresses, e.g., hepatocytes.

Traditionally, in the field of evolutionary biology, the preservation and occurrence of polyploidy preserved across the tree of life have been attributed to the idea that WGD contributes to genome stability. The increased DNA content may protect the cell and the organism from mutations or chromosomal aberrations as a result of DNA damage (121–123) (Table 1). If left unpurged or unrepaired, the inexorable accumulation of deleterious genetic events has been termed Muller’s ratchet. Indirect evidence for this argument can be found in the many redundant paralogs of multiple genes and pathways observed in the genomes of higher organisms (75, 123–125). Essentially, the presence of an extra copy of chromosomes serves as a genetic backup system to protect against the effects of mutations and DNA strand breaks that would otherwise cause a cell to undergo apoptosis and be lost from the tissue pool (126). WGD has also been proposed as a mechanism to increase genome instability to allow organisms to generate more heritable variation in response to environmental stress (74, 127–130). The increase in DNA material also correlates with increased cell size, suggesting that WGD may be a strategy for growth of either the cell, the tissue, or the organism.

The diverse functional roles of polyploidy across evolution and development testify to the scope of cell enlargement reaching beyond genome size or stability (121, 122, 124). Indeed, cell enlargement may allow a cell to perform more functions independently, as opposed to adhering to and dividing labor among multiple cells. Greater functionality and independence can be achieved only by polyploidization. Cell enlargement provides, for example, increased amounts of biomolecules to be utilized for nutrients, for increased organelle function, or to mitigate the effects of environmental change. Endoreplication efficiently provides the nutrients and materials needed to support developing eggs or embryos, such as *Drosophila* follicle and nurse cells and mammalian trophoblasts (31). The biomass of polyploid cells in fruit fly larvae provides nutrients during larval feeding (31) and plants use increased cell size to increase nutrient storage (86). Cell enlargement for enhancing organelle functions results from increased gene dosage and subsequent downstream machinery for the increased production of cellular building blocks, including RNA, proteins, and lipids as well as increased energy production though increased numbers of mitochondria (88, 92). Polyploidization provides an economy of scale for cell metabolic processes.

While genetic programming also relates to long-term adaptions, cells adapt to oxidative stress in the short term by metabolic reprograming (80). In mammals, increased machinery for platelet production is facilitated by megakaryocytes that increase intracellular materials and metabolism by serially doubling their genomes and inhibiting cytokinesis (76, 78, 131). Finally, cell enlargement that mitigates the effects of environmental change is observed in, for example, protists and plants. One group of protists, the Foraminifera, toggle between haploidy, diploidy, and polyploidy during harsh and toxic conditions in oceans, lakes, and soil (132–134). During temporary decreases of light or water, polyploid leaf and root cells maximize surface area via endoreplication to maximize surface area and uptake (135). Plant seedlings also utilize polyploidy for fast growth out of the dark soil and into the light. During injury, salamander cells and mammalian liver cells can regenerate functional tissue by accessing polyploid programs, even when cell division is blocked (76). It is noteworthy that polyploid cells facilitate development and survival during harsh conditions not only by their increased genomic material, but by their increased cellular contents and sheer size (127, 128).

## Benefits to Polyaneuploidy for Tumor Survival: Predictions

The generation of WGD and consequent polyploidy and aneuploidy are well documented in the cancer literature (67, 136). PACCs as a cancer cell life history state can have multiple non-mutually exclusive advantages that act in a cooperative manner across the cancer cell population within a tumor (137, 138). This polyploidization appears to be a necessary step to induce a pause in the cell cycle to protect the cell from stress-induced DNA damage. Since loss of contact inhibition and concomitant uncontrolled proliferation is a hallmark of cancer, accessing a polyploid program after DNA replication may be the only mechanism available for cancer cells to exit the cell cycle (65). We predict that 2N+ cancer cells do not exit the cell cycle to a nonproliferative state unless they become PACCs. Further work will define the interrelationships between PACCs, quiescence, senescence, and cell cycle control.

Increased genomic material, such as is observed in PACCs, has been noted in evolutionary and developmental biology. Being a 4N+ cell provides several potential advantages, including increased genetic stability to prevent apoptosis in a cell with damaged DNA as well as the generation of beneficial mutations to promote survival of resistant progeny (Table 1) (76, 79, 139). PACCs eventually undergo depolyploidization to produce “typical” non-PACC progeny and repopulate tumor sites through cell division, amitotic mechanisms (e.g., neosis), or both (16, 34, 37, 91, 92, 140, 141). The timing of PACC depolyploidization is relevant to understanding cancer recurrence through the generation of proliferative progeny as well as the exit from apparent “dormancy” (or paused proliferation) often associated with cancer metastasis (142). Understanding the dynamics of polyploidization, quiescence of PACCs, and depolyploidization may also shed light on how these structurally abnormal cells evade recognition and subsequent destruction by the immune system (22, 25, 61). In our own experiments, when PACCs generate progeny, we do not observe generation of multiple nonviable cells as would be suggested by a genetic instability mechanism (29, 99). Their 2N+ progeny have increased resistance to different forms of stress. We predict that PACCs do not utilize genetic instability as the mechanism to generate progeny with therapeutic resistance. Further work will define the role of genome protection through quiescence versus mutation in the generation of progeny and population rescue of tumors.

The increased cell size of PACCs is not in dispute, but the functional purpose for this increase in cellular machinery has not been established (Table 1) (8, 16, 20, 23, 34, 35, 143). Evolutionary and developmental polyploidization programs utilize the additional genomic material to provide the building blocks for increased cell metabolism. It is possible that the increased cell size allows PACCs to store more energy molecules (e.g., lipids, proteins, carbohydrates) and to survive extended periods of dormancy. It is also possible that increased cell size (with its concomitant decreased surface-to-volume ratio) provides protection from toxin and oxidative stress via increased production of RNA and protein for protective pathways. We predict that PACCs have altered metabolism as compared to 2N+ cancer cells that is shunted toward cell survival and detoxification while downregulating cell proliferation pathways. Further work will characterize the mechanisms that contribute to PACC survival under stress as well as the mechanisms that release PACCs from quiescence and reenter the cell cycle to begin proliferation.

## Conclusions

We present a unifying theory to explain cancer recurrence and lethality. The hallmarks of cancer provide the framework for tumorigenesis and are complemented by the hallmark of therapeutic resistance that is enabled by polyaneuploidy that enables recurrence and lethality (39). Specifically, therapeutic resistance enabled by the access of a polyploidization program represents an additional hallmark of cancer, representing a “hallmark of lethal cancer.” Access to these evolutionary and developmental programs allows a paused cell cycle and concomitant WGD, further enabling subsequent cellular capabilities of sustained dormancy and genetic modification that result in resistant progeny and tumor regrowth.

## Data Availability

There are no data underlying this work.
